# miR-100 Suppresses the Proliferation, Invasion, and Migration of Hepatocellular Carcinoma Cells via Targeting CXCR7

**DOI:** 10.1155/2021/9920786

**Published:** 2021-07-17

**Authors:** Yiman Ge, Jia Shu, Gang Shi, Fuguo Yan, Yejing Li, Hangliang Ding

**Affiliations:** ^1^Department of Clinical Laboratory, Hospital of Chengdu University of Traditional Chinese Medicine, Chengdu 610072, China; ^2^Department of Functional Inspection Division, Hospital of Chengdu University of Traditional Chinese Medicine, Chengdu 610072, China; ^3^Department of Pharmacy Services, Hospital of Chengdu University of Traditional Chinese Medicine, Chengdu 610072, China; ^4^Department of General Surgery, The Xinchang Hospital Affiliated to Wenzhou Medical University, Xinchang 312501, China; ^5^Department of Clinical Laboratory Center, Central Hospital of Enshi Autonomous Prefecture, Enshi 445001, China

## Abstract

This study is to elucidate the functions of miR-100 in hepatocellular carcinoma progression and to explore the underlying mechanisms. Expression levels of miR-100 in normal-cancer hepatocellular carcinoma tissues were measured using quantitative real-time PCR (qRT-PCR). The invasive and proliferative abilities of hepatocellular carcinoma cell lines transfected with mimic-NC or mimic-miR-100 were measured using transwell, CCK-8, and colony formation assays. The binding sites between CXCR7 and miR-100 were determined using luciferase reporter assays. The correlation of CXCR7 and miR-100 in hepatocellular carcinoma progression was further confirmed by cotransfection assays. Our results showed that miR-100 was significantly lower expressed in hepatocellular carcinoma tissues and negatively associated with CXCR7 expression. Cell functional assays' results found that upregulation of miR-100 inhibited the proliferative, invasive, and migrative abilities in hepatocellular carcinoma cells. Luciferase reporter assay suggested that CXCR7 mRNA and miR-100 bound one another. Increasing CXCR7 expression reversed the inhibitive effects of upregulated miR-100 in hepatocellular carcinoma cells. Further study showed that miR-100/CXCR7 played a role in hepatocellular carcinoma progression by regulating metalloproteinase-2 (MMP2) and vascular endothelial growth factor (VEGF). Conclusively, miR-100 exerts antitumor effects on hepatocellular carcinoma. Overexpression of miR-100 attenuates the invasive and proliferative abilities of hepatocellular carcinoma cells by targeting CXCR7.

## 1. Introduction

Cancer has threatened public health for a long time, and liver cancer is one type of malignant tumors with high mortality [1, 2]. As the main subtype of liver cancer, hepatocellular carcinoma accounts for 75-85% approximately, much more than other types [1]. Furthermore, the morbidity of hepatocellular carcinoma trends to increase in the future [3]. Although tremendous advances have been completed in the therapy for hepatocellular carcinoma, there is no significant decline in the death rate of hepatocellular carcinoma patients because of the frequently occurred tumor metastasis [4]. The increased detection rate of hepatocellular carcinoma has improved patients' survival [5]. Nevertheless, it remains difficult to treat hepatocellular carcinoma diagnosed at advanced stages, and the survival rate for these patients remains low. Thus, it is imperative to explore the molecular mechanisms underlying hepatocellular carcinoma progression to find novel strategies for treatment.

MicroRNAs (miRNAs), containing less than 22 nucleotides, are the main type of noncoding RNAs (ncRNAs) and can modulate the progression of various cancers. Increasing studies have reported that numerous functional miRNAs are dysregulated in tumor tissues and cell lines and exert crucial effects in the initiation and progression of various tumors. For example, low expression of miR-329 and miR-362-3p exerts an oncogenic effect in human breast cancer [6]. Given that downregulation of Y-box binding protein 1 (YBX1) played an inhibitive role in malignant pleural mesothelioma (MPM), miR-137 can suppress MPM progression by targeting YBX1 [7]. The vital roles played by miRNAs in hepatocellular carcinoma progression were also reported by many studies. miR-206 can bind to the 3′-UTR of the cMET gene to downregulate its expression and further exert antitumor functions in hepatocellular carcinoma [8]. miR-302b is dramatically downregulated in hepatocellular carcinoma tissues, and overexpression of miR-302b can promote the proliferation and invasion of hepatocellular carcinoma cells [9].

Among all these miRNAs, miR-100 is a well-studied one. miR-100 has been found to exert an antitumor function in some cancers, including esophageal squamous cancer [10], colorectal cancer [11], breast cancer [12], and nasopharyngeal carcinoma [13]. Besides, the antitumor role of miR-100 was also observed in hepatocellular carcinoma [14–16]. However, the underlying mechanisms of miR-100 in hepatocellular carcinoma progression have not been reported. Thus, we aim to explore the expression and function of miR-100 in hepatocellular carcinoma and analyze the underlying mechanism in this present study.

## 2. Materials and Methods

### 2.1. Clinical Samples

In our present study, a total of 42 paired specimens, including cancerous tissues and the matched normal tissues from the same hepatocellular carcinoma patients, were collected. The diagnosis of hepatocellular carcinoma was confirmed by postoperative pathology. Before the collection of these specimens, no participant has received radiotherapy or chemotherapy. Written consents have been provided by all patients, and the Ethics Committee of Central Hospital of Enshi Autonomous Prefecture approved this study.

### 2.2. Cell Culture and Mice

The HEK293 cells and hepatocellular carcinoma cell line LM3 were purchased from the Shanghai Cell Bank. Dulbecco's Modified Essential Medium (DMEM), supplemented with 10% foetal bovine serum (FBS) and 100 *μ*g/ml penicillin/streptomycin, was used to incubate these cells. A special incubator with a temperature of 37°C and 5% CO_2_ was utilized to incubate target cells. BALB/C nude mice (28–35 days old) were provided by the Animal Core Facility (Shanghai, China) and housed at 25°C under pathogen-free conditions.

### 2.3. Plasmid Construction and Transfection

The miR-100 precursor (mimic) was provided and transferred into pcDNA6.2-GW/EmGFP-miR vector by Genepharm company (Hangzhou, Zhejiang, China). A miRNA with the sequence of “5′-AGGTACGAAACGCTAAGAAT-3′” was used as the control [10]. The guidelines of Lipofectamine 2000 (Invitrogen, Carlsbad, CA, USA) were used to perform transfection. LM3 cell line was transfected with pcDNA6.2/miR-100 to construct overexpressing miR-100 cell line. Similarly, the full-length CXCR7 sequence was subcloned into pBabe-CXCR7 retroviruses vectors, provided by Genepharm company (Hangzhou, Zhejiang, China), to construct overexpressing CXCR7 LM3 cell line. All transfected cells were selected with 2.0 *μ*g/ml puromycin for 7 days.

### 2.4. qRT-PCR

Total RNA was extracted from tissues of hepatocellular carcinoma patients and the control healthy persons and LM cells using TRIzol reagent (Invitrogen, USA), based on its instruction. Reversing 1 *μ*g total RNA to cDNA was done using the NCode™ VILO™ miRNA cDNA synthesis kit (Invitrogen). qRT-PCR was performed following its manufacturer's protocol using SYBR II Premix Taq (Takara, Japan). U6 mRNA was applied as the endogenous reference. The PCR primers were purchased from Sangon Company (Shanghai, China).

Several computational prediction databases (PicTar, TargetScan, and miRanda) were used to screen the downstream targets of miR-100. CXCR7 which contains a putative target sequence of miR-100 in 3′-UTR was identified. To investigate the expressed association between miR-100 and CXCR7, the expression levels of CXCR7 were also analyzed with primers as follows: forward primer: 5′-GGCTATGACACGCACTGCTACA-3′ and reverse primer: 5′-TGGTTGTGCTGCACGAGACT-3′. The sequences of other specific primers were as follows: Ki-67 forward primer: 5′-AAT TCAGACTCCATGTGCCTGAG-3′ and reverse primer: 5′-CTTGAC ACACACATTGTCCTCAGC-3′; proliferating cell nuclear antigen (PCNA) forward primer: 5′-CACCTTAGCACTAGTATT CGAAGCAC-3′ and reverse primer: 5′-CACCCGACGGCATCT TTATTAC-3′. *β*-Actin was applied as an endogenous reference with the used primers: *β*-actin forward 5′-AGAAAATCTGGCACCACACC-3′ and *β*-actin reverse: 5′-TAGCACAGCCTGGATAGCAA-3′. Finally, the expression levels of target genes were calculated by the 2^-*ΔΔ*Ct^ method.

### 2.5. CCK-8 Assay

The CCK-8 assay was performed to evaluate the promotive ability of LM3 cells with or without transfection. Briefly, LM3 cells were placed into a 96-well plate with a density of 2000 cells/well and cultured with DMEM, added with 10% FBS. The OD value was measured to evaluate proliferation using Cell Counting Kit-8 (CCK-8), while a microplate reader (PerkinElmer, USA) was used to measure the OD value of LM3 cells at 450 nm.

### 2.6. Invasion and Migration Assays

The invasion of LM3 hepatocellular carcinoma cells transfected with miR-NC or miR-100 mimics was examined by transwell assays with 8 *μ*m pore chambers (Corning, NY, USA), precoated with Matrigel (BD, USA) in the upper chamber. First, 1 × 10^5^ target cells were transferred into an upper chamber and incubated with a serum-free medium. The lower chamber was added with 20% FBS supplemented medium to play the role of chemoattractant. After that, noninvading cells were removed with a cotton swab, while invasive LM3 cells were stained with hematoxylin-eosin. Finally, invasive LM3 cells were counted by a light microscope.

Wound healing assays were carried out to assess the migrative ability of transfected LM3 cells. Target cells were seeded into 6-well plates and cultured up to 80-90% confluence. After that, a 20 *μ*l pipette tip was used to scratch a straight line on the bottom. After culturing for 24 hours in an incubator at 37°C, target cells were observed using an optical microscope to measure the migration distance.

### 2.7. Flow Cytometry

The target cells (2 × 10^6^) were collected and fixed in 70% ethanol for 12 hours. After that, these cells were washed three times with PBS and the supernatant was discarded. Then, we added 100 *μ*l RNase ribonuclease I (100 *μ*g/ml) into the centrifuge tubes for 30 min at 37°C.

Subsequently, propidium iodide (PI) was added and cultured at 20°C for 10 min. The samples were transferred into Falcon tubes and analyzed by FACScan, and the CellQuest software (BD Biosciences) was used to calculate the percentages of cells occupying the G0/G1, S, and G2/M phases.

### 2.8. Luciferase Reporter Assay

The mutant-type and wild-type vectors of pMIR-CXCR7-3′-UTR were purchased from GenePharma (Hangzhou, China). LM3 hepatocellular carcinoma cells were cotransfected with mimic-miR-100 or mimic-NC and CXCR7 mutant-type or wild-type vector. After 48 hours of incubation at 37°C, luciferase activity of these cells was measured by a Dual-Luciferase Assay Kit (Promega, Madison, Wisconsin) based on its manufacturer's protocol.

### 2.9. Western Blot

Total proteins from overexpressing miR-100 or the control LM3 cells were extracted using radioimmunoprecipitation assay lysis buffer (KeyGen Biotech, Nanjing, China) based on the manufacturer's instructions. A bicinchoninic acid protein assay kit (KeyGen Biotech, Nanjing, China) was used to determine protein concentrations. Subsequently, electrophoresis was carried out to separate proteins, and then, the proteins were transferred onto polyvinylidene fluoride (PVDF) membranes (Millipore, Billerica, MA, USA) at 300 mA for 1.2 h. Membranes were then blocked using 5% skim milk for 1.5 h at 20°C. They were incubated into primary antibodies overnight at 4°C. Primary antibodies included CXCR7 (1 : 1000, Cell Signaling Technology, Danvers, USA), Ki-67 (1 : 1000, Abcam Inc., Cambridge, MA, USA), PCNA (1 : 1000, Abcam Inc., Cambridge, MA, USA), VEGF (1 : 1000, Abcam Inc., Cambridge, MA, USA), MMP2 (1 : 1000, Abcam Inc., Cambridge, MA, USA), and GAPDH (1 : 1000, Santa Cruz). Thereby, the membranes were incubated with anti-mouse secondary antibodies for 1 hour at 20°C. Signals were examined using a chemiluminescence detection kit (Thermo Fisher, USA) after these membranes were washed by TBS-T (10 min/time) three times.

### 2.10. Subcutaneous Metastasis Model in Nude Mice

Randomly, 12 mice were separated into two groups. miR-NC- or miR-100-transfected LM3 cells were seeded into the subcutaneous area of the necks of the nude mice. The tumors were dissected from the mice, and the tumor volumes were measured from 12 days to 27 days at 3-day intervals. Tumor volume was calculated by the formula (height × width × length)/2. The Institutional Committee for Animal Research approved the animal experiments.

### 2.11. Statistical Analysis

Data analysis was performed using SPSS 19.0 software (IBM, Chicago, IL, USA), and the results were expressed as means ± standard deviation. Significant differences between the two groups were analyzed by Student's *t*-test. *p* < 0.05 was determined as a statistical significance.

## 3. Results

### 3.1. miR-100 Was Downregulated in Hepatocellular Carcinoma Tissues and Negatively Associated with CXCR7

**Figure 1:**
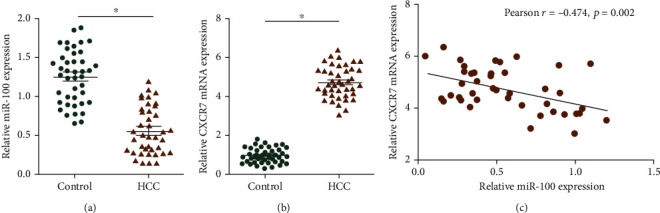
Expression levels of miR-100 and CXCR7 in hepatocellular carcinoma tissues and the control: (a) miR-100; (b) CXCR7; (c) Pearson analysis. ^∗^*p* < 0.05.

The expression levels of miR-100 and CXCR7 were detected in 42 paired hepatocellular carcinoma and the matched normal tissues using qRT-PCR. The results showed that the expression levels of miR-100 in hepatocellular carcinoma tissues were significantly lower than those in the control group, whereas CXCR7 expression was higher in cancerous tissues (Figures 1(a) and 1(b)). Further, Pearson analysis found that there was a negative correlation between miR-100 and CXCR7 expression (Figure 1(c)).

### 3.2. miR-100 Suppressed Hepatocellular Carcinoma Cell Proliferation, Invasion, and Migration

**Figure 2:**
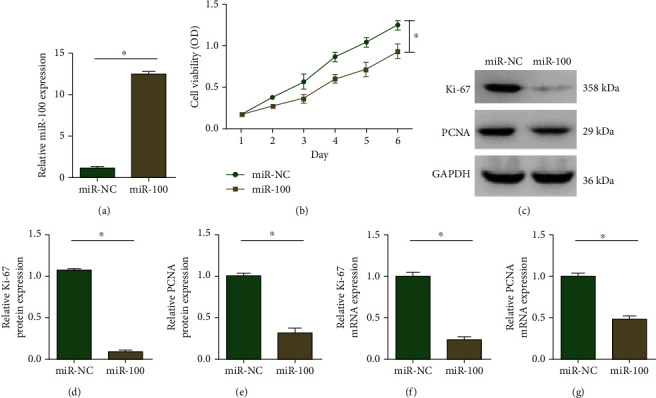
Upregulation of miR-100 suppressed hepatocellular carcinoma cell proliferation. (a) Relative expression levels of miR-100 in LM3 cells transfected with miR-NC or miR-100 mimics. (b) CCK-8 results showed that miR-100 overexpression inhibited the proliferation of hepatocellular carcinoma cells. (c–g) Western blot and qRT-PCR assays found that miR-100 overexpression reduced the protein and mRNA levels of Ki-67 and proliferating cell nuclear antigen (PCNA). ^∗^*p* < 0.05.

LM3 cells were transfected with miR-100 mimics to construct miR-100-overexpressing cell lines, while the control group was transfected with empty plasmid. Subsequently, qRT-PCR was performed and a higher expression of miR-100 was observed in the miR-100 mimic group (Figure 2(a)). Then, CCK-8 assays were performed and the results (Figure 2(b)) showed that miR-100 upregulation inhibited the proliferation of LM3 hepatocellular carcinoma cells. The proliferation-inhibitory effect of miR-100 was further confirmed by cell proliferation markers, Ki-67 and PCNA. The results of western blot and qRT-PCR showed that miR-100 overexpression reduced the protein (Figures 2(b)–2(d)) and mRNA (Figures 2(e) and 2(f)) levels of Ki-67 and PCNA.

**Figure 3:**
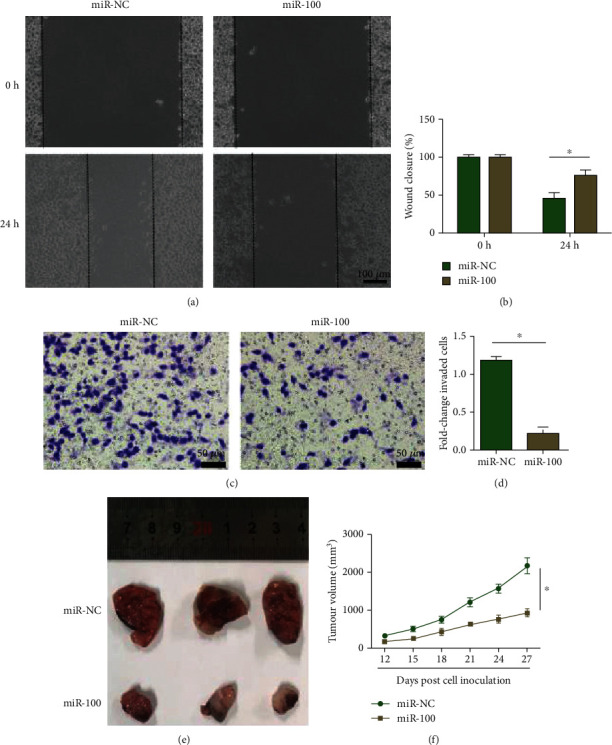
Upregulation of miR-100 suppressed hepatocellular carcinoma cell invasion, migration, and growth in vivo. (a, b) Wound healing assay showed that miR-100 overexpression inhibited the migration of hepatocellular carcinoma cells. (c, d) Transwell assay showed that miR-100 overexpression inhibited the invasion of hepatocellular carcinoma cells. (e, f) miR-100 overexpression suppressed the growth in vivo of hepatocellular carcinoma cells. ^∗^*p* < 0.05.

Besides, the effects of miR-100 on the migration and invasion of LM3 cells were also examined by wound healing and transwell assays and the results showed miR-100 upregulation inhibited the migration (Figures 3(a) and 3(b)) and invasion (Figures 3(c) and 3(d)) of LM3 hepatocellular carcinoma cells. Furthermore, nude mice were implanted subcutaneously with transfected LM3 cells to investigate whether miR-100 suppresses LM3 cell growth in vivo. The results demonstrated that miR-100 overexpression suppresses the tumorigenesis of hepatocellular carcinoma in vivo.

### 3.3. miR-100 Targets CXCR7

**Figure 4:**
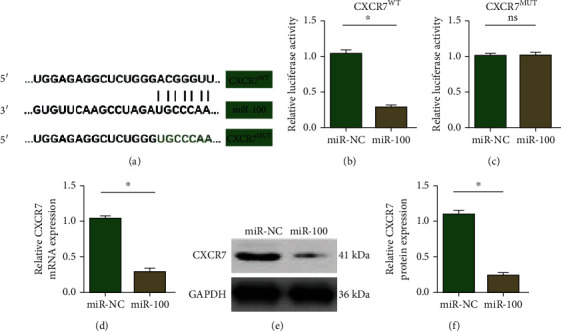
miR-100 directly targets CXCR7. (a) The binding sites between miR-100 and CXCR7. (b, c) Luciferase activity assay between wild-type CXCRT 3′-UTR or mutation-type CXCRT 3′-UTR and miR-100. (d) Relative CXCR7 mRNA expression in LM3 cells after overexpressing miR-100. (e, f) Relative CXCR7 protein expression in LM3 cells after overexpressing miR-100. ^∗^*p* < 0.05.

PicTar, TargetScan, and miRanda databases were used to investigate the downstream target of miR-100. Among these predicted proteins, we observed that there were binding sites between miR-100 and CXCR7 (Figure 4(a)). After that, we performed a luciferase assay to confirm the direct binding sequences between miR-100 and CXCR7 3′-UTR region and the results showed that luciferase activity was significantly decreased in 3′-UTR wild type of CXCR7 (Figures 4(b) and 4(c)). After transfecting with miR-100 mimics in LM3 cells, relative CXCR7 mRNA and protein levels were examined, and the results suggested that CXCR7 was markedly reduced in mRNA and protein levels after upregulating miR-100 expression (Figures 4(d)–4(f)). All of these results suggested that CXCR7 was a direct target of miR-100.

### 3.4. miR-100 Suppressed Hepatocellular Carcinoma Progression via Modulating CXCR7

**Figure 5:**
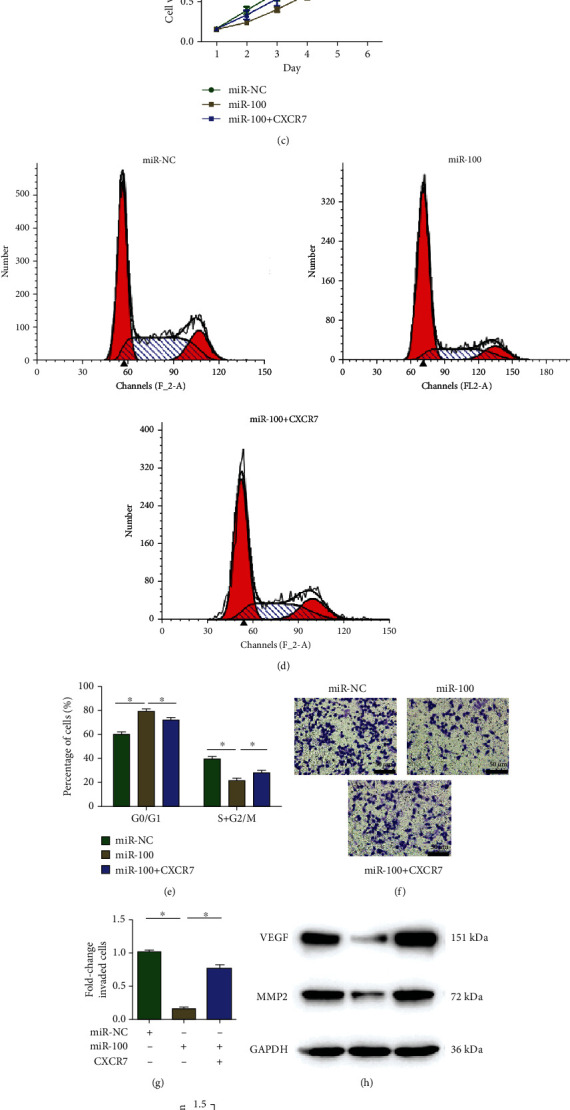
CXCR7 partially reverses the effects of overexpressing miR-100 on LM3 cells. (a, b) Relative CXCR7 protein expression in LM3 cells after transfection of miR-100 or cotransfection of miR-100 and CXCR7. (c) CCK-8 results showed that CXCR7 overexpression partially reversed the inhibitive effects of miR-100 on LM3 cell proliferation. (d, e) miR-100 upregulation arrested the G0/G1 to S phase transition in LM3 cells, while CXCR7 exerted reverse effects. (f, g) Transwell assay showed that CXCR7 overexpression partially reversed the inhibitive effects of miR-100 on LM3 cell invasion. (h, i) miR-100 upregulation reduced the protein levels of VEGF and MMP2, while CXCR7 increased their expression. ^∗^*p* < 0.05.

To confirm whether miR-100 exerted antitumor functions in hepatocellular carcinoma by targeting CXCR7, cotransfection of miR-100 mimics and CXCR7-overexpressing vector was performed in LM3 cells. After transfection of the CXCR7-overexpressing vector, the protein levels of CXCR7 were markedly increased (Figures 5(a) and 5(b)). Furthermore, CCK-8 assay results showed that CXCR7 overexpressing partially reversed the suppressive effects of miR-100 on LM3 cell proliferation (Figure 5(c)). Next, the cell cycle was analyzed to determine whether miR-100 inhibited cell proliferation by altering the cell cycle. The results demonstrated that upregulation of miR-100 altered the cell cycle by arresting the G0/G1 to S phase transition, while CXCR7 overexpressing partially reversed this alteration (Figures 5(d) and 5(e)). Besides, a transwell assay was performed to analyze the role of miR-100/CXCR7 in hepatocellular carcinoma cell invasion. The results showed that increasing CXCR7 expression could reverse the suppressive effects of miR-100 on LM3 cell invasion. The expression levels of two invasive markers, VEGF and MMP2, were examined by western blot assay, and the results showed that miR-100 upregulation reduced the protein levels of VEGF and MMP2 (Figures 5(f) and 5(g)), whereas reverse effects were observed after overexpressing CXCR7 (Figures 5(h) and 5(i)). Taken together, these findings suggested that miR-100 suppressed hepatocellular carcinoma cell proliferation and invasion by targeting CXCR7.

## 4. Discussion

In the present study, we found miR-100 was downregulated in hepatocellular carcinoma tissues and exerted antitumor functions on hepatocellular carcinoma progression via regulating CXCR7. Over the past decades, miRNA has attracted the growing attention of researchers as the posttranscriptional regulators and numerous studies have verified that miRNA plays a vital role in disease development, including cancer [17, 18]. For example, miR-342-3p inhibits the initiation and progression of hepatocellular carcinoma by regulating MTC1 [19]. miR-199a-3p inhibits the oncogenesis of hepatocellular carcinoma and hepatic apoptosis via targeting programmed cell death 4 [20]. As a newly identified miRNA, miR-100 also regulated tumor initiation and progression. For example, Peng and colleagues found that miR-100 suppressed the proliferation and induced apoptosis of gastric cancer cells by targeting BMPR2 [21]. However, there are no reports that revealed the specific role and mechanism between miR-100 expression and the biological progression of hepatocellular carcinoma up to date. The role of miR-100 in regulating hepatocellular carcinoma and the potential molecular mechanism might provide a novel direction in the study of hepatocellular carcinoma progression.

CXCR7, also termed as named receptor dog cDNA 1 (RDC1), was the seventh reported of chemokine receptor family CXC class [22, 23]. Chemokine receptors are responsible for the running status of various singling pathways in cell biology [24], and a growing amount of studies has suggested that chemokines and their receptors exert important functions in the growth, angiogenesis, lymphatic metastasis, and osseous metastasis of tumors. For example, expression levels of CXCR3, CXCR4, and CXCR5 displayed a marked increase in gastric cancer and were significantly correlated to the prognosis of patients [25]. CXCR7 can induce the activation of the MAPK signaling pathway and then lead to resistance to second-generation antiandrogen therapy in prostate cancer [26]. In hepatocellular carcinoma, CXCR7 can also promote tumor progression by activating MAPK signaling [27]. Moreover, recent studies and bioinformatic databases identified that CXCR7 was an important downstream target of miR-100. For example, miR-100 suppressed the proliferation of gastric cancer cells by binding to the 3′-untranslated region of CXCR7 [28]. Besides, the inhibitive role of the miR-100/CXCR7 axis was also observed in esophageal squamous cancer and ductal carcinoma [10, 29].

Then, we detected the expression levels of miR-100 and CXCR7 in 42 paired hepatocellular carcinoma and the matched normal tissues using qRT-PCR. The results revealed that miR-100 had a low level in hepatocellular carcinoma tissues and was negatively associated with CXCR7 expression. To further explore the roles of miR-100/CXCR7, we constructed miR-100-overexpressing cell lines of hepatocellular carcinoma and performed a series of functional experiments. The results showed overexpressed miR-100 weakened the proliferative and invasive abilities of hepatocellular carcinoma cells. Mechanistically, miR-100 exerted anticancer effects by targeting CXCR7. We found that miR-100 suppressed LM3 cell proliferation by altering the cell cycle, which could be reversed by simultaneous upregulation of CXCR7. Besides, miR-100 overexpression reduced protein levels of VEGF and MMP2, whereas the reverse effects were observed in CXCR7 upregulation.

Although our results well revealed the antitumor role of the miR-100/CXCR7 axis in hepatocellular carcinoma, there were several issues that need to be improved in our future study. First, more hepatocellular carcinoma tissues and control tissue samples should be used to confirm the miR-100 expression levels in hepatocellular carcinoma. Second, miR-100 silencing in hepatocellular carcinoma cells should be performed and subsequent changes in biological behaviors of hepatocellular carcinoma cells should be examined. Third, the roles of the miR-100/CXCR7 axis in hepatocellular carcinoma should be reconfirmed in more cell lines.

## 5. Conclusions

This study suggested that miR-100 can function as a tumor suppressor by inhibiting the proliferation, migration, and invasion of LM3 hepatocellular carcinoma cells. miR-100 suppressed hepatocellular carcinoma progression by targeting CXCR7. Therefore, the miR-100/CXCR7 axis might become an attractive therapeutic target for hepatocellular carcinoma treatment.

## Data Availability

Data in our study can be acquired by emailing the corresponding author upon reasonable request.

## References

[1] Bray F., Ferlay J., Soerjomataram I., Siegel R. L., Torre L. A., Jemal A. (2018). Global cancer statistics 2018: GLOBOCAN estimates of incidence and mortality worldwide for 36 cancers in 185 countries. *CA: A Cancer Journal for Clinicians*.

[2] Zhou M., Wang H., Zeng X. (2019). Mortality, morbidity, and risk factors in China and its provinces, 1990-2017: a systematic analysis for the Global Burden of Disease Study 2017. *The Lancet*.

[3] Forner A., Reig M., Bruix J. (2018). Hepatocellular carcinoma. *The Lancet*.

[4] Forner A., Gilabert M., Bruix J., Raoul J. L. (2014). Treatment of intermediate-stage hepatocellular carcinoma. *Nature Reviews. Clinical Oncology*.

[5] Couri T., Pillai A. (2019). Goals and targets for personalized therapy for HCC. *Hepatology International*.

[6] Kang H., Kim C., Lee H. (2016). Downregulation of microRNA-362-3p and microRNA-329 promotes tumor progression in human breast cancer. *Cell Death and Differentiation*.

[7] Johnson T. G., Schelch K., Cheng Y. Y. (2018). Dysregulated expression of the microRNA miR-137 and its target YBX1 contribute to the invasive characteristics of malignant pleural mesothelioma. *Journal of Thoracic Oncology*.

[8] Wang Y., Tai Q., Zhang J. (2019). MiRNA-206 inhibits hepatocellular carcinoma cell proliferation and migration but promotes apoptosis by modulating cMET expression. *Acta Biochimica et Biophysica Sinica*.

[9] Wang L., Yao J., Zhang X. (2014). miRNA-302b suppresses human hepatocellular carcinoma by targeting AKT2. *Molecular Cancer Research*.

[10] Zhou S. M., Zhang F., Chen X. B. (2016). miR-100 suppresses the proliferation and tumor growth of esophageal squamous cancer cells via targeting CXCR7. *Oncology Reports*.

[11] Lampropoulou D. I., Aravantinos G., Laschos K., Theodosopoulos T., Papadimitriou C., Gazouli M. (2019). MiR-218 and miR-100 polymorphisms as markers of irinotecan-based chemotherapy response in metastatic colorectal cancer. *International Journal of Colorectal Disease*.

[12] Wu G., Zhou W., Pan X. (2018). miR-100 reverses cisplatin resistance in breast cancer by suppressing HAX-1. *Cellular Physiology and Biochemistry*.

[13] Sun X., Liu X., Wang Y., Yang S., Chen Y., Yuan T. (2018). miR-100 inhibits the migration and invasion of nasopharyngeal carcinoma by targeting IGF1R. *Oncology Letters*.

[14] Cai J. P., Zhou X. H., Yu H. B., Li D. Y., Zhou B. X. (2020). Study on the clinical significance of miR-100 expression in the invasion and metastasis of hepatocellular carcinoma. *Zhonghua Gan Zang Bing Za Zhi*.

[15] Petrelli A., Perra A., Schernhuber K. (2012). Sequential analysis of multistage hepatocarcinogenesis reveals that miR-100 and PLK1 dysregulation is an early event maintained along tumor progression. *Oncogene*.

[16] Chen P., Zhao X., Ma L. (2013). Downregulation of microRNA-100 correlates with tumor progression and poor prognosis in hepatocellular carcinoma. *Molecular and Cellular Biochemistry*.

[17] Goodall G. J., Wickramasinghe V. O. (2021). RNA in cancer. *Nature Reviews Cancer*.

[18] Rupaimoole R., Calin G. A., Lopez-Berestein G., Sood A. K. (2016). miRNA deregulation in cancer cells and the tumor microenvironment. *Cancer Discovery*.

[19] Komoll R.-M., Hu Q., Olarewaju O. (2021). MicroRNA-342-3p is a potent tumour suppressor in hepatocellular carcinoma. *Journal of Hepatology*.

[20] Li Z., Zhou Y., Zhang L. (2020). microRNA-199a-3p inhibits hepatic apoptosis and hepatocarcinogenesis by targeting PDCD4. *Oncogenesis*.

[21] Peng C. W., Zhou Y. Q., Tang S. (2019). miR-100-3p inhibits cell proliferation and induces apoptosis in human gastric cancer through targeting to BMPR2. *Cancer Cell International*.

[22] Tarnowski M., Liu R., Wysoczynski M., Ratajczak J., Kucia M., Ratajczak M. Z. (2010). CXCR7: a new SDF-1-binding receptor in contrast to normal CD34(+) progenitors is functional and is expressed at higher level in human malignant hematopoietic cells. *European Journal of Haematology*.

[23] Naumann U., Cameroni E., Pruenster M. (2010). CXCR7 functions as a scavenger for CXCL12 and CXCL11. *PLoS One*.

[24] Eva C., Sprengel R. (1993). A novel putative G protein-coupled receptor highly expressed in lung and testis. *DNA and Cell Biology*.

[25] Yu C., Zhang Y. (2019). Characterization of the prognostic values of CXCR family in gastric cancer. *Cytokine*.

[26] Li S., Fong K. W., Gritsina G. (2019). Activation of MAPK signaling by CXCR7 leads to enzalutamide resistance in prostate cancer. *Cancer Research*.

[27] Lin L., Han M. M., Wang F., Xu L. L., Yu H. X., Yang P. Y. (2014). CXCR7 stimulates MAPK signaling to regulate hepatocellular carcinoma progression. *Cell Death & Disease*.

[28] Cao Y., Song J., Ge J., Song Z., Chen J., Wu C. (2018). MicroRNA-100 suppresses human gastric cancer cell proliferation by targeting CXCR7. *Oncology Letters*.

[29] Shams R., Seifi-Alan M., Bandehpour M., Omrani M. D., Ghafouri-Fard S. (2020). C-X-C chemokine receptor type 7 (CXCR-7) expression in invasive ductal carcinoma of breast in association with clinicopathological features. *Pathology Oncology Research*.

